# Heavy Metal-Based Nanoparticles as High-Performance X-ray Computed Tomography Contrast Agents

**DOI:** 10.3390/ph16101463

**Published:** 2023-10-15

**Authors:** Mohammad Yaseen Ahmad, Shuwen Liu, Tirusew Tegafaw, Abdullah Khamis Ali Al Saidi, Dejun Zhao, Ying Liu, Sung-Wook Nam, Yongmin Chang, Gang Ho Lee

**Affiliations:** 1 Department of Chemistry, College of Natural Sciences, Kyungpook National University, Taegu 41566, Republic of Korea; yaseen.knu@gmail.com (M.Y.A.); liushuwen0701@gmail.com (S.L.); tegafawtirusew@yahoo.com (T.T.); abdullah_al_saidi@hotmail.com (A.K.A.A.S.); djzhao.chem@gmail.com (D.Z.); ly1124161@gmail.com (Y.L.); 2 Department of Molecular Medicine, School of Medicine, Kyungpook National University, Taegu 41944, Republic of Korea; nams@knu.ac.kr

**Keywords:** heavy metal-based nanoparticles, X-ray attenuation, contrast agents, in vitro phantom imaging, in vivo imaging

## Abstract

X-ray computed tomography (CT) contrast agents offer extremely valuable tools and techniques in diagnostics via contrast enhancements. Heavy metal-based nanoparticles (NPs) can provide high contrast in CT images due to the high density of heavy metal atoms with high X-ray attenuation coefficients that exceed that of iodine (I), which is currently used in hydrophilic organic CT contrast agents. Nontoxicity and colloidal stability are vital characteristics in designing heavy metal-based NPs as CT contrast agents. In addition, a small particle size is desirable for in vivo renal excretion. In vitro phantom imaging studies have been performed to obtain X-ray attenuation efficiency, which is a critical parameter for CT contrast agents, and the imaging performance of CT contrast agents has been demonstrated via in vivo experiments. In this review, we focus on the in vitro and in vivo studies of various heavy metal-based NPs in pure metallic or chemical forms, including Au, Pt, Pd, Ag, Ce, Gd, Dy, Ho, Yb, Ta, W, and Bi, and provide an outlook on their use as high-performance CT contrast agents.

## 1. Introduction

Heavy metal-based nanoparticles (NPs) are extremely valuable materials in various applications due to their high surface-to-volume ratios and optical, magnetic, antibacterial, and mechanical properties [1,2,3]. Surface modification can endow them with biocompatibility and colloidal stability for various biomedical applications, such as imaging, cancer therapy, and targeted drug delivery. In particular, they can strongly attenuate X-rays because X-ray attenuation is proportional to atomic number (Z) [4]. Therefore, heavy metal-based NPs can serve as contrast agents in X-ray computed tomography (CT) [5,6,7,8,9,10,11].

Contrast agents play a key role in CT as they enable the accurate diagnosis and monitoring of diseases via contrast enhancements [5]. In addition, they allow for the detection of small lesions by increasing contrast. Heavy metal-based NPs have emerged as a promising class of powerful CT contrast agents as they possess a high density of heavy metal atoms with high X-ray attenuation coefficients.

Conventional iodine (I, Z = 53)-based CT contrast agents, which are organic compounds with three iodines per molecule or six iodines per dimeric molecule [12], have shown limitations such as short blood circulation times and high injection doses (>1 mmol/kg) due to their low imaging sensitivity. High injection doses can cause renal toxicity or side effects [13]. In addition, iodine-based CT contrast agents are randomly dispersed in the intravascular and extravascular regions, resulting in confounding CT images [14]. Heavy metal-based NPs can be used to overcome the limitations of iodine-based CT contrast agents due to their higher X-ray attenuation and longer blood circulation times. Furthermore, heavy metal-based NPs can easily be surface-modified with targeting molecules to enhance the discernibility of specific diseases, organs, and tissues, making them excellent candidates for highly sensitive and specific CT contrast agents [15,16].

Biocompatibility, colloidal stability, and renal excretion must be carefully designed to develop heavy metal-based NPs as CT contrast agents [17,18,19]. Surface modification can be used to improve biocompatibility and colloidal stability. In particular, polyethylene glycol (PEG) can be used to improve blood circulation times [20,21,22]. The elimination of NPs via the renal system is essential because most heavy metals are toxic [23]. This can be achieved via reducing the particle diameter (d) and hydrodynamic diameter (HD) (<3 nm) [18,19].

Various heavy metal-based NPs have been investigated as potential CT contrast agents in pure metallic or chemical forms, including palladium (Pd, Z = 46) [24], silver (Ag, Z = 47) [25,26,27,28], cerium (Ce, Z = 58) [29,30], gadolinium (Gd, Z = 64) [31,32,33,34,35], dysprosium (Dy, Z = 66) [32,36,37], holmium (Ho, Z = 67) [37,38,39,40], ytterbium (Yb, Z = 70) [32,41,42,43,44,45,46], tantalum (Ta, Z = 73) [32,47,48,49,50], tungsten (W, Z = 74) [51,52,53,54,55], platinum (Pt, Z = 78) [56,57,58,59,60,61,62,63], gold (Au, Z = 79) [64,65,66,67,68,69,70,71,72,73,74,75,76,77,78,79,80,81], and bismuth (Bi, Z = 83) [32,82,83,84,85,86,87,88] (Table 1). These NPs are overviewed in this paper, and their performances are compared with those of commercial iodine-based CT contrast agents based on their X-ray attenuation efficiencies and in vivo performance.

## 2. Basic Principles of CT Contrast Agents

**Figure 1:**
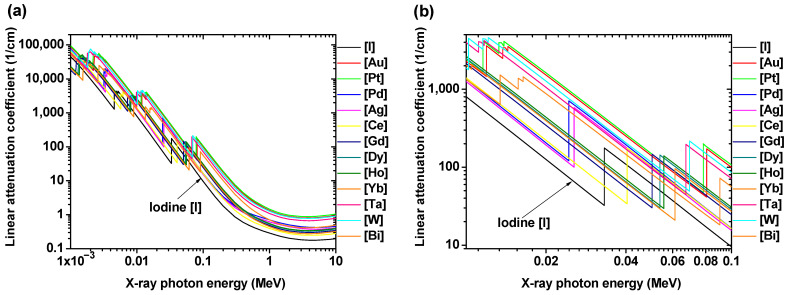
Linear X-ray attenuation coefficients [4] of heavy metal atoms and I versus X-ray photon energy between (**a**) 1 keV and 10 MeV and (**b**) 10 and 100 keV (clinical energy area).

CT is a medical imaging technique that provides detailed cross-sectional images of the body using the distinct X-ray attenuation properties of tissues, bones, organs, and blood vessels [89]. In particular, CT is useful for imaging bones and hardened diseases because soft tissues made of light elements (C, H, O, N, etc.) weakly attenuate X-rays. As shown in Figure 1a,b, the linear X-ray attenuation coefficients of heavy metal atoms are higher than that of iodine [4], making their NPs more effective CT contrast agents than iodine-based CT contrast agents.

CT and magnetic resonance imaging (MRI) are the most widely used clinical imaging techniques which provide high-spatial-resolution (~1 mm) and three-dimensional anatomical images with high penetration depths suitable for whole body imaging [90]. In addition, they can be used to diagnose various conditions, including tumors, fractures, and internal bleeding. CT offers faster scanning than MRI, but its sensitivity to the imaging probe is less than that of MRI [90], requiring approximately ten times higher injection doses for iodine contrast agents compared with gadolinium contrast agents in MRI. This high injection dose of iodine-based CT contrast agents may be a burden to patients [91], which can be overcome using heavy metal-based NPs with increased imaging sensitivity.

## 3. Heavy Metal-Based CT Contrast Agents

### 3.1. Noble Metal-Based NPs

Noble metal-based NPs can exist in pure metallic forms (Pt, Pd, Ag, and Au) or, occasionally, in chemical forms. They also exhibit photothermal properties useful for cancer therapy [92].

#### 3.1.1. Pd (Z = 46)-NPs

Pd-based NPs also possess high photothermal conversion efficiency and high photothermal stability, making them promising for applications in nanomedicine, such as cancer imaging and treatment.

**Figure 2:**
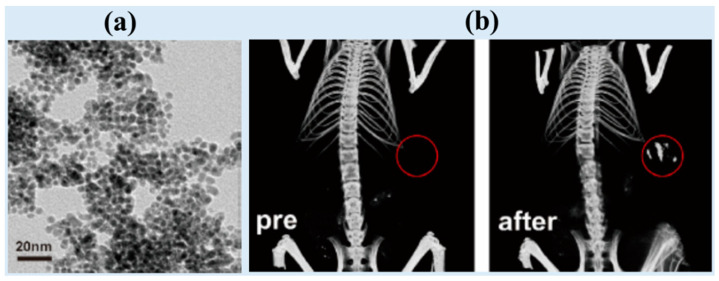
(**a**) TEM image of cysteamine-coated FePd bimetallic nanodots. (**b**) In vivo CT images at the tumor site (red circles) of mice before (labeled as “pre”) and 12 h after intravenous injection [24].

Lyu et al. synthesized cysteamine-coated FePd bimetallic nanodots (d = 3.4 nm) (Figure 2a) as trimodal CT-MRI-photoacoustic (PA) imaging agents [24]. FePd bimetallic nanodots were synthesized using NaBH_4_ as a reducing agent in a nitrogen atmosphere and then coated with cysteamine for colloidal stability and biocompatibility via sonication in ethanol. They obtained an X-ray attenuation efficiency of 2.6 HU/mM, higher than that of the commercial iodine contrast agent Iopromide (2.0 HU/mM). In addition, high contrast enhancement was observed in the in vivo CT images of BALB/c mice at the tumor site (red circles in Figure 2b) 12 h after intravenous injection (dose = 50 μL, 10 mg Pd/mL) (Figure 2b). The nanodots offered excellent CT signal augmentation and showed exceptional stability and biocompatibility. In addition, they were applied to in vivo thermoradiotherapy (photothermal therapy combined with radiation therapy).

#### 3.1.2. Ag (Z = 47)-NPs

Among noble metal NPs, Ag-NPs have attracted interest due to their novel optical (i.e., surface plasmon resonance), photothermal, and antimicrobial properties. In addition, Ag-NPs have received considerable interest in medical imaging as potential CT contrast agents due to their high X-ray attenuation properties.

Liu et al. synthesized generation 5 (G5) dendrimer-stabilized Ag-NPs (Ag-DSNPs) with particle diameter control (d = 8.8, 12.4, 16.1, and 23.2 nm) [25]. Amine-terminated G5- poly(amidoamine) dendrimers were used to encapsulate Ag-NPs for colloidal stability and biocompatibility. The X-ray attenuation efficiency (η) depended on the particle diameter, meaning that η (~7.2 HU/mM; 16.1 nm) > η (~4.6 HU/mM; 12.4 nm) > η (~4.0 HU/mM; 23.2 nm) > η (~2.4 HU/mM; 8.8 nm) at 80 kVp. The η value of the 16.1 nm Ag-NPs was similar to that of the commercial iodine contrast agent Omnipaque. Moreover, Ag-DSNPs (d = 16.1 nm) exhibited contrast enhancements at the injection site in vivo (dose = 50 μL, [Ag] = 0.1 M), whereas Omnipaque did not because of its fast diffusion due to its low molecular weight. The injected Ag-DSNPs were nontoxic; thus, mice injected with Ag-DSNPs were healthy and exhibited normal behavior.

**Figure 3:**
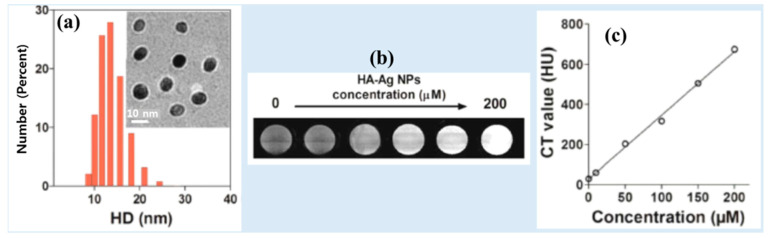
(**a**) Histogram of the hydrodynamic diameter (HD) distribution of HA-Ag-NPs based on dynamic light scattering (DLS) and cryo-TEM (inset). (**b**) Phantom images as a function of Ag concentration. (**c**) CT values (i.e., X-ray attenuation) of HA-Ag-NPs as a function of Ag concentration [26].

Zhang et al. synthesized spherical and monodispersed hyaluronic acid-coated Ag-NPs (HA–Ag-NPs) (d = ~10 nm, HD = 13.5 nm) through one-pot NaBH_4_ reduction (Figure 3a) [26]. The HA-Ag-NPs exhibited excellent long-term stability in water and low cytotoxicity. The in vitro phantom images demonstrated the strong X-ray attenuation of HA-Ag-NPs, which increased with increasing Ag concentrations (Figure 3b,c). The NPs exhibited an X-ray attenuation coefficient of 3.5 kHU/mM.

Hsu et al. synthesized glutathione-conjugated Ag_2_S NPs using a viscosity-mediated, thermal decomposition method at 165 °C in a nitrogen atmosphere with size control (d = 2.3, 3.1, 4.1, and 5.1 nm) based on the reaction time [27]. Because the NPs were ultrasmall, they were renally excretable. For example, 85% of the injected 3.1 nm glutathione-conjugated Ag_2_S NPs were excreted via the renal system with urine. The X-ray attenuation efficiency was nearly independent of the particle diameter and decreased with increasing X-ray tube voltages so that 80 kVp (~3.5 HU/mM) > 100 kVp (~2.7 HU/mM) > 120 kVp (~2.3 HU/mM) > 140 kVp (~1.9 HU/mM). In addition, the X-ray attenuation efficiency was lower than that of the commercial iodine contrast agent Iopamidol (~5.1 HU/mM at 80 kVp, ~4.3 HU/mM at 100 kVp, ~3.4 HU/mM at 120 kVp, and ~3.0 HU/mM at 140 kVp). The potential of Ag_2_S NPs as CT contrast agents was demonstrated in vivo by observing contrast enhancements at the bladder, heart, and kidneys after intravenous injection (dose = 250 mg Ag/kg). In vivo CT imaging indicated NP excretion with urine with minor accumulation in the liver and spleen. Therefore, the ultrasmall Ag_2_S NPs with efficient renal excretion exhibited great potential for clinical translation.

Cui et al. synthesized bovine serum albumin (BSA)-stabilized Ag nanodots (d = 5.8 nm) using biomineralization, in which a mixture of BSA and AgNO_3_ in a basic aqueous solution was stirred. The BSA-stabilized Ag nanodots exhibited an X-ray attenuation efficiency of 5.7 HU/mM, higher than that of the commercial iodine contrast agent Iopromide (4.3 HU/mM) [28]. The NPs were intratumorally injected (injection dose = 10 mg/mL, 20 μL), and contrast enhancements at the mice tumor site were observed after injection and then decreased with time, demonstrating their potential as a CT contrast agent. In addition, the signal intensities of the bladder and the tumor gradually decreased, proving that the BSA-stabilized Ag nanodots were renally excretable. Furthermore, the authors demonstrated the efficiency of the BSA-stabilized Ag nanodots in photothermal therapy of tumors in vivo using an 808 nm near infrared laser after intratumoral injection.

#### 3.1.3. Pt (Z = 78)-NPs

Pt with a high atomic number (Z = 78) possesses a high X-ray attenuation coefficient that is useful for CT imaging [4]. In addition, it has the PA effect, which can be used in PA imaging, and a high photothermal conversion efficiency, which is useful for photothermal and radiation based on cancer therapies.

Wang et al. developed an albumin-mediated one-pot synthesis method to generate ultrasmall Pt@BSA NPs for CT imaging by reducing H_2_PtCl_6_ using BSA as the biotemplate and NaBH_4_ as the reducing agent in an aqueous solution [56]. The Pt@BSA NPs with a core size of 2.1 nm showed excellent colloidal stability, hemocompatibility, and biocompatibility. The X-ray attenuation efficiency of the Pt@BSA NPs (16.8 HU/mM) at 120 kVp was approximately 2.4 times higher than that of the commercial iodine contrast agent Ultravist (~6.3 HU/mM). The contrast enhancement of the heart was observed 5 min after the intravenous injection of the Pt@BSA NPs, whereas no contrast enhancement was observed at the heart after the intravenous injection of Ultravist, indicating the enhanced CT imaging capabilities of the Pt@BSA NPs compared to those of iodine-based CT contrast agents. In addition, the contrast of the heart, which plays an important role in vascular imaging, gradually decreased over 4 h before stabilizing to usual levels, indicating the extended angiographic capabilities of the Pt@BSA NPs in vivo.

**Figure 4:**
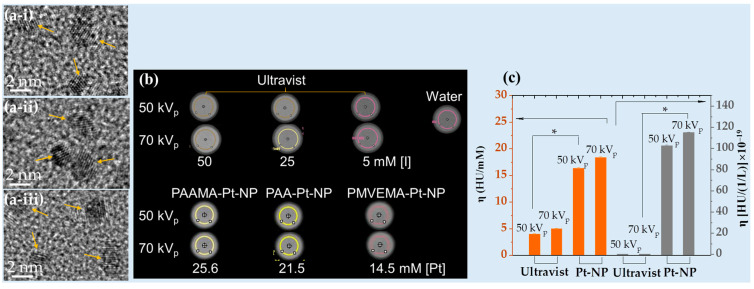
(**a**) TEM images of (i) PAA-, (ii) PAAMA-, and (iii) PMVEMA-coated Pt-NPs. The arrows indicate NPs. (**b**) Phantom images at 50 and 70 kVp. (**c**) Histogram of X-ray attenuation efficiency. *: *p* < 0.05, the significance of the results was confirmed using a *t*-test. The commercial iodine contrast agent Ultravist was used as a reference [57].

Recently, Saidi et al. designed Pt-NPs coated with hydrophilic and biocompatible polymers, namely poly(acrylic acid) (PAA), poly(acrylic acid-co-maleic acid) (PAAMA), and poly(methyl vinyl ether-alt-maleic acid) (PMVEMA) (d = 2.0 nm, Figure 4a), using a one-pot polyol method. The polymer-coated Pt NPs exhibited approximately four times higher X-ray attenuation efficiencies (i.e., 16.4 HU/mM at 50 kVp and 18.4 HU/mM at 70 kVp) at the same atomic concentration (Figure 4b,c) [57], and approximately 500 times higher X-ray attenuation efficiency at the same density compared to Ultravist (Figure 4c).

Zhang et al. synthesized Ce6-PEG-coated mesoporous Pt (mPt) nanoplatforms (Pt@PEG-Ce6) (Ce6 = photosensitizer chlorin e6, d = 70 nm, and pore diameter = 11 nm) [58]. The mPt nanomaterials were synthesized by reducing the Pt precursor in an aqueous solution, followed by Ce6 coating for colloidal stability, biocompatibility, and photosensitization. Pt@PEG-Ce6 exhibited an X-ray attenuation efficiency of 3.1 HU/mM at 120 kVp. In addition, the contrast at the tumor site significantly increased after intravenously injecting 100 mL of Pt@PEG-Ce6 (1 mg/mL) into the mice, indicating that the NPs can be used as a CT contrast agent. In addition, further studies were conducted on the biosafety of Pt@PEG-Ce6 in vivo. The biochemical parameters of blood were assessed 24 h after the intravenous injection of Pt@PEG-Ce6 (1 mg/mL) into BALB/c mice. The results showed that Pt@PEG-Ce6 had no visible toxicity as the blood biochemical indicators of the injected mice were not significantly different from those of healthy mice. In addition, Pt@PEG-Ce6 was applied to CT/PA imaging-guided photodynamic therapy (PT) of cancer in vivo.

Fu et al. synthesized mesoporous Pt-NPs (mesoPt-NPs) using Pluronic F127 as a structure-directing agent without seeds or organic reagents [59]. The synthesized mesoPt-NPs showed a spherical structure and uniform diameter (94 nm). The surface of the mesoPt-NPs was modified with PEG, and the prepared mesoPt-PEG NPs showed excellent biocompatibility and an X-ray attenuation efficiency of 5.547 HU/mM at 120 kVp. In addition, the authors loaded the mesoPt-PEG NPs with doxorubicin (Dox; anticancer drug) and performed chemo-photothermal therapy of cancer cells in vitro, where 84% of cancer cells were killed.

Ma et al. synthesized poly(maleic anhydride-alt-1-octadecene)–PEG-coated (or simply PEGylated) Pt nanoworms (HD = ~100 nm) [60]. The Pt nanoworms were synthesized using thermal decomposition in a nitrogen atmosphere and then coated with C18PMH-PEG for colloidal stability and biocompatibility. The Pt-PEG nanoworms exhibited an X-ray attenuation efficiency of 3.9 HU/mM. In addition, they observed contrast enhancements at the tumor site after subcutaneous injection (dose = 16 mg/kg). In addition, the Pt-PEG nanoworms exhibited extensive blood circulation due to their PEG chains [20,21,22], demonstrating that they passively targeted tumors via the enhanced permeability and retention (EPR) effect. The authors also applied the Pt-PEG nanoworms to CT/PA imaging-guided photothermal/radiation therapy of cancers in vivo.

Jameel et al. synthesized highly stable, biocompatible, and ultrasmall Pt-NPs with a mean diameter of 3.8 nm using a one-step, one-pot, eco-friendly, and simple process [61]. They used an extract from Prosopis farcta fruits as a reducing agent and stabilizer and obtained an X-ray attenuation efficiency of 6.6 HU/mM at 80 kVp, higher than that of the commercial iodine contrast agent Omnipaque (3.1 HU/mM).

Tang et al. synthesized human serum albumin (HSA)-coordinated monodisperse 6.7 nm Pt-NPs by reducing the Pt precursor with NaBH_4_ in an aqueous solution, followed by reaction with HSA [62]. The synthesized HSA-coordinated Pt-NPs exhibited an X-ray attenuation efficiency of ~5.6 HU/mM, which was higher than that of Iopromide (~5.2 HU/mM). The HSA-coordinated Pt-NPs were intratumorally injected at 250.0 μmol Pt/kg to evaluate their potential for in vivo CT imaging, and they demonstrated higher contrast enhancements at the tumor site than those obtained with Iopromide due to the higher X-ray attenuation coefficient of Pt compared to that of iodine and the excellent tumor retention of the NPs. In addition, the authors successfully applied the HSA-coordinated Pt-NPs to CT/PA dual imaging and photothermal cancer therapy in vivo.

Wang et al. synthesized hollow Pt cubes (or spirals) with an average size of 30 nm via the selective etching of Pd@Pt core–shell nanostructures using HCl, followed by modification with PEG-SH for biocompatibility [63]. With an X-ray attenuation efficiency of 5.39 HU/mM, Pt spirals offered higher contrast than that of Omnipaque (4.76 HU/mM). Their superiority as a CT contrast agent was demonstrated via in vivo imaging, which showed significant contrast enhancement at the tumor site 24 h after intravenous injection (a 1.45-fold increase against preinjection). The enhanced contrast at the tumor was attributed to the EPR effect of the NPs due to the long blood circulation time (the measured circulation halftime = 2.91 h). In addition, the Pt spirals exhibited outstanding photothermal properties that exceeded those of Pt NPs, making them useful for in vivo photothermal cancer therapy.

#### 3.1.4. Au (Z = 79)-NPs

Au-NPs are noble metal-based NPs that have been intensively investigated as CT contrast agents due to the superior X-ray attenuation ability [4] and biocompatibility of Au. In addition, Au-NPs possess photothermal properties applicable to cancer therapy [92,93]. Owing to their high affinity to thiol derivatives, Au-NPs can be easily surface modified to improve their biocompatibility, colloidal stability, and durability.

Dong et al. generated six PEGylated Au-NPs with various particle diameters (4, 15, 50, 79, 100, and 152 nm; HD = 24.1, 40.7, 69.9, 96.9, 104.8, and 140.6 nm, respectively). For the synthesis of 4 and 15 nm Au-NPs, the gold precursor was reduced using NaBH_4_, and for the larger Au-NPs, a seed growth method was used. The authors found that X-ray attenuation did not depend on the Au-NP size (4.0–4.2 HU/mM at 80 kVp) [78]. After intravenous injection (injection dose = 500 mg Au/kg), they observed that the smaller Au-NPs (4 nm and 15 nm) provided CT contrasts with a longer circulation time compared to the larger Au-NPs because mononuclear phagocytic systems find it more difficult to remove them as they pass through the liver and kidneys, leading to the accumulation of a large number of small Au-NPs in the blood. On the other hand, large Au-NPs (>50 nm) accumulated in the liver and spleen, providing excellent CT contrast in those areas. This particle size-dependent NP biodistribution suggests that NPs with appropriate particle sizes must be used to achieve the intended diagnostic and therapeutic applications.

Tsvirkun et al. coated Au-NPs with diameters of 10, 30, and 100 nm (Cytodianostic Inc., Burlington, ON, Canada) with PEG-COOH and then GB111-NH_2_ via the amide bond [79]. Then, targeting ligand cathepsin was conjugated with GB111-PEG-Au-NPs. The cathepsin-labeled Au-NPs exhibited an X-ray attenuation efficiency of 25.2 HU/mM at 35 kVp and 22.0 HU/mM at 85 kVp, slightly higher than those of the iodine contrast agent (5.4 and 4.0 HU/mM, respectively). In addition, enhanced in vivo CT imaging signals were observed at the tumor site using 10 and 30 nm Au-NPs rather than 100 nm Au-NPs due to the more efficient diffusion of smaller NPs after intravenous injection. For tumor targeting, they used the enzyme targeting method. In addition, the authors observed that tumor-targeted imaging exhibited higher contrast enhancements than nontargeted (or passive) imaging for all particle sizes and that the targeted imaging contrast decreased with increasing particle size, indicating that smaller NPs (10–100 nm) can provide better contrast in targeted imaging.

**Figure 5:**
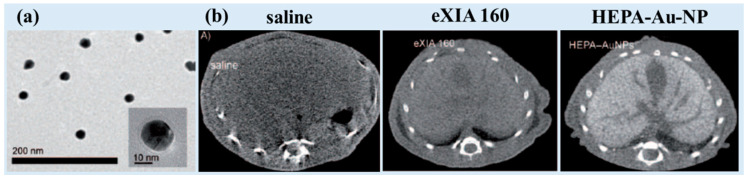
(**a**) TEM image of HEPA–Au-NPs. (**b**) Cross-sectional in vivo micro-CT images of the liver 2 h after the injection of saline, eXIA 160 (800 mg I/kg), and HEPA-Au-NPs (250 mg Au/kg) [80].

Sun et al. synthesized the heparin–amino acid 3,4-dihydroxyphenylalanine (DOPA)- conjugated Au-NPs (HEPA-Au-NPs) (d = 24.0 nm; HD = 54.6 nm). They coated Au-NPs (British Biocell International, Salisbury, UK) with HEPA in an aqueous solution, leading to low toxicity and sustained stability [80]. They employed the HEPA-Au-NPs as a liver-specific CT imaging agent. The average size of the heparin coating layers on the surface was approximately 20 nm, almost the size of the bare Au-NPs, as can be observed using TEM (Figure 5a). The HEPA-Au-NPs provided 21.9 HU/mM at 70 kVp, whereas the commercial iodine contrast agent eXIA 160 provided 4.2 HU/mM. In vivo micro-CT images revealed that the HEPA-Au-NPs produced enhanced liver-specific CT images compared with iodine-based contrast agents (Figure 5b). Approximately 2 h after injection, HEPA-Au-NPs showed highest contrast in the liver, which was 12.6 and 3.2 times higher than that of saline and eXIA 160, respectively. In addition, the efficient liver targeting of the HEPA-Au-NPs was confirmed from the liver-specific contrast, which persisted for up to 24 h. This nanoplatform has potential as a molecular imaging probe and liver-specific CT imaging agent for the monitoring of liver cancer.

Hainfeld et al. used 1.9 nm Au-NPs (Nanoprobes, Inc., preparation # 1101, Yaphank, NY, USA) for in vivo mice experiments [81]. The injection dose of the Au-NPs suspended in phosphate-buffer saline (PBS) at pH 7.4 was 0.01 mL/g for the Au-NP solution (concentration = 270 mg Au/mL). After intravenous injection via mice tails, high-resolution CT images of blood vessels (<100 µm) were obtained, whereas they were not obtained using the iodine contrast agent Omnipaque. In addition, kidneys and tumors were observed via high-spatial-resolution images. No evidence of toxicity was observed from blood plasma analytes and organ histology at both 11 days and 30 days after intravenous injection.

### 3.2. Lanthanide (Ln)-Based NPs

Ln has a higher X-ray attenuation coefficient than iodine (I) [4]; thus, it can be used in CT contrast agents. In addition, Ln exhibits optical and magnetic properties, enabling the development of multimodal imaging agents.

#### 3.2.1. Ce (Z = 58)-Based NPs

Ce-based NPs possess various properties useful for biomedical applications, such as high X-ray attenuation [4] and antioxidant properties [94]. Therefore, Ce-based NPs can be employed in the imaging of anatomical features with enhanced contrast and protection against X-ray radiation damage during CT because Ce can effectively remove reactive oxygen species formed during a CT scan.

**Figure 6:**
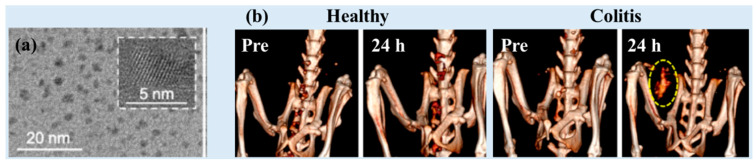
(**a**) TEM images of Dex-Ce-NPs (inset: 5 nm scale). (**b**) CT images of healthy (left) and colitis (right) mice obtained before (labeled as “pre”) and 24 h after the oral administration of Dex-Ce-NPs. The yellow dashed circle indicates CT contrast as Dex-Ce-NPs accumulated in the colitis-affected region 24 h after injection [29].

Naha et al. used a precipitation method to synthesize dextran-coated CeO_2_ NPs (Dex-Ce-NPs) (i.e., precipitation of cerium salts after addition to ammonium hydroxide in the presence of dextran) (d = 4.8 nm and HD = 17.5 nm) (Figure 6a) as CT contrast agents for gastrointestinal tract (GIT) imaging [29]. The Dex-Ce-NPs exhibited X-ray attenuation efficiencies of ~6.3 HU/mM (80 kVp), ~4.8 HU/mM (100 kVp), ~3.8 HU/mM (120 kVp), and ~3.2 HU/mM (140 kVp). The Dex-Ce-NPs exhibited no contrast in the large intestines of healthy mice, whereas they exhibited contrast in the colitis-affected region (Figure 6b). The dextran coating on the NP surfaces provided stability in aqueous media, biocompatibility, and specificity toward colitis. More than 97% of the oral doses were cleared from the body within 24 h. In addition, the Dex-Ce-NPs helped to reduce oxidative damage induced by CT scans by scavenging free radicals generated due to the ionization of X-ray radiation, implying that Dex-Ce-NPs can be used as a potential CT contrast agent for imaging GIT with colitis.

García et al. synthesized albumin-stabilized 5.1 nm CeO_2_ NPs via the chemical precipitation of a Ce^3+^ precursor in a basic aqueous solution using tetramethylammonium hydroxide, followed by the dropwise addition of a CeO_2_ NP solution to the albumin solution [30]. The NPs exhibited contrast enhancements in the liver, whereas the commercial iodine contrast agent Iopamidol-370 exhibited fast accumulation in the kidneys, followed by renal excretion. A tenfold contrast enhancement was obtained compared to those of commercial contrast agents. Efficient uptake by the liver and spleen was observed up to 7 days after intravenous tail injection, and 85% of the injected dose was recovered. In addition, the intratumoral injection of the albumin-stabilized CeO_2_ NPs led to contrast enhancements at the tumor site for up to 7 days after injection, and 99% of the injected dose remained at the tumor site, allowing for the monitoring of tumor growth and dynamics.

#### 3.2.2. Gd (Z = 64)-Based NPs

Trivalent Gd(III) possesses the highest spin magnetic moment (s = 7/2) among the elements in the periodic table. Therefore, Gd(III)-chelates have been commercially developed as positive (T_1_) MRI contrast agents [91]. In addition, the X-ray attenuation coefficient of Gd is higher than that of iodine [4], implying that Gd-based NPs are potential candidates for MRI-CT dual imaging agents.

**Figure 7:**
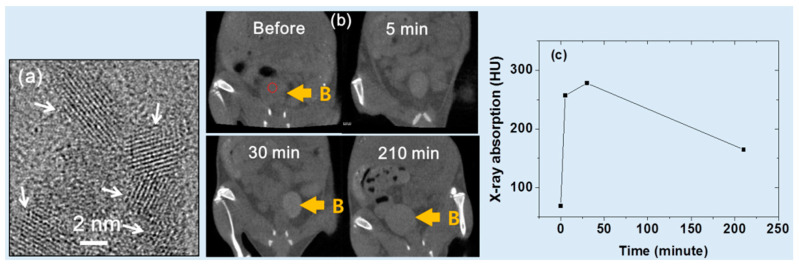
(**a**) HRTEM image of ultrasmall Gd_2_O_3_ NPs coated with the iodine compound (i.e., C_8_H_4_I_3_NO_4_) (indicated by the arrows). (**b**) In vivo CT images of a mouse bladder (marked B) at 70 kVp. The red circle indicates region of interest (ROI). (**c**) X-ray absorption of the ROI of the bladder (indicated by the small dotted circle) (B) before and after the intravenous injection of an aqueous NP sample into the mouse’s tail [31].

Ahmad et al. [31] developed iodine compound (i.e., C_8_H_4_I_3_NO_4_)-coated ultrasmall Gd_2_O_3_ NPs with a mean diameter of ~2 nm using a one-pot polyol method (Figure 7a). The iodine compound was used as a surface-coating ligand as it is hydrophilic, biocompatible, and can boost X-ray contrast via the use of iodine. They observed an X-ray attenuation efficiency of 11.8 HU/mM at 70 kVp. Following intravenous tail injection (injection dose = ~0.53 mmol Gd/kg), brighter contrast enhancements were observed for the mouse bladder (indicated by B in Figure 7b). The contrast at the region-of-interest (ROI) of the bladder, denoted by the small dotted circle in Figure 7b, peaked at approximately 30 min after injection and then gradually reduced over time (Figure 7c), indicating that the sample solution was expelled through the bladder as urine. As a result, the potential of iodine compound-coated Gd_2_O_3_ NPs as a CT contrast agent was demonstrated. In addition, they observed high positive contrasts in MR images in vivo, demonstrating the T_1_ MRI-CT dual imaging ability of the NPs.

Ghazanfari et al. examined the X-ray attenuation characteristics of PAA-coated ultrasmall Gd_2_O_3_ NPs with an average particle diameter of 1.9 nm, which were synthesized using the one-pot polyol method [32]. The hydrophilic and biocompatible PAA was used for surface coating to ensure good colloidal stability and nontoxicity. The samples showed excellent biocompatibility and colloidal stability. The PAA-coated Gd_2_O_3_ NPs demonstrated higher X-ray attenuation (5.9 HU/mM) than Ultravist (4.40 HU/mM) at 70 kVp.

Zheng et al. synthesized PAA-capped GdF_3_ NSs (10.6 × 7.0 × 4.2 nm) as bimodal MRI-CT contrast agents [35]. In this study, GdF_3_ nanoplates were synthesized using the thermolysis of Gd^3+^ precursors in high-boiling-point nonpolar solvents in a nitrogen atmosphere and then coated with PAA via the ligand exchange method. The NSs exhibited ~7.9 HU/mM at 60 kVp, which was higher than that of the commercial iodine contrast agent Iohexol (~4.8 HU/mM).

#### 3.2.3. Dy (Z = 66)-Based NPs

Dy possesses a high X-ray attenuation coefficient [4]. Therefore, Dy-based NPs can strongly attenuate X-rays, leading to high contrast in CT images. In addition, Dy exhibits a high magnetic moment (10.4–10.6 Bohr magneton) at room temperature; thus, Dy-based NPs can be used as negative (T_2_) MRI-CT dual imaging agents.

Recently, Ghazanfari et al. synthesized PAA-coated ultrasmall Dy_2_O_3_ NPs with an average particle diameter of 1.8 nm using the one-pot polyol method and characterized their X-ray attenuation properties [32]. The NPs exhibited excellent colloidal stability in aqueous media and nontoxicity in vitro due to the PAA coating. They observed an X-ray attenuation efficiency of 6.1 HU/mM at 70 kVp for the Dy_2_O_3_ NP suspension, greater than that of Ultravist (4.4 HU/mM).

Olifirenko et al. synthesized polyethyleneimine (PEI)-coated Dy_2_O_3_ NPs (d = 79–102 nm) in aqueous media [36]. They prepared Dy_2_O_3_ NPs via calcination; subsequently, the NPs were coated with PEI in an aqueous solution. The PEI-coated Dy_2_O_3_ NPs precipitated in 6 h, but could be redispersed via shaking; they exhibited low cellular toxicity up to 100 μg/mL due to the PEI coating. The authors observed an X-ray attenuation efficiency of ~5 HU/mM at 120 kVp.

Gómez-Gónzalez et al. synthesized PAA-coated DyVO_4_ NPs (d = 60 nm and HD = 81 nm) using a polyol method [37]. They observed an X-ray attenuation efficiency of 4.8 HU/mM at 65 kVp, higher than that of Iohexol (1.6 HU/mM).

#### 3.2.4. Ho (Z = 67)-Based NPs

Similar to Dy, Ho possesses a high X-ray attenuation coefficient [4]; thus, Ho-based NPs can be used as CT contrast agents. In addition, the high magnetic moment of Ho (10.4–10.7 Bohr magneton) at room temperature enables the development of Ho-based NPs as T_2_ MRI-CT dual imaging agents.

**Figure 8:**
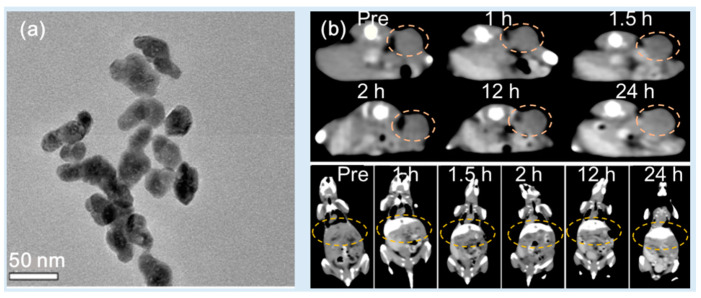
(**a**) TEM image of PEG-HoF_3_ NPs. (**b**) In vivo CT images of tumor-bearing Kunming mice showing bright contrast enhancements at the tumor site (top) and liver (bottom) for 24 h after intravenous tail injection [38]. The dotted circles indicate the tumor (top) and the liver (bottom).

Zhang et al. synthesized PEG-modified HoF_3_ NPs using a one-pot solvothermal technique [38]. The PEG-HoF_3_ NPs exhibited size uniformity and exhibited excellent dispersibility in an aqueous solution due to the PEG coating (d = 38 nm and HD = ~100 nm) (Figure 8a). The X-ray attenuation efficiency was 19.0 HU/mM at 120 kVp, which was higher than that of Iohexol (3.0 HU/mM). The PEG-HoF_3_ NPs demonstrated good biocompatibility and low toxicity in histological studies and a cytotoxicity evaluation. For in vivo CT images, the PEG-HoF_3_ NP solution was injected into tumor-model Kunming mice tail veins (dose = 100 μL, 2 mg/mL). The distribution of the PEG-HoF_3_ NPs was monitored using CT at different time points. An enhanced brightness of the tumor site and liver was observed for 24 h after injection (Figure 8b). However, the kidneys did not show obvious contrast enhancements because the large NP size blocked renal filtration. This may reduce kidney side effects.

Ni et al. synthesized PEGylated NaHoF_4_ NPs with various particle diameters (d = 3.2, 7.4, and 13.2 nm; HD = 12.9, 19.0, 22.7 nm) and NRs (28.9 × 16.7 nm) by controlling the ligand concentration, temperature, and time [40]. They obtained an X-ray attenuation efficiency of 6.9 HU/mM at 120 kVp for 3.2 nm PEGylated NaHoF_4_ NPs, higher than that of the commercial iodine contrast agent Iobitridol (2.1 HU/mM). They observed enhanced contrast at the thighs of the Kunming mice after directly injecting the 3.2 nm PEGylated NaHoF_4_ NPs into the thigh (injection dose = ~120 μg Ho). In addition, the NPs exhibited a five-fold contrast increase at the tumor after injection compared with that before injection (injection dose = ~80 μg Ho).

Gómez-Gónzalez et al. synthesized PAA-coated HoVO_4_ NPs (d = 65 nm and HD = 74 nm) using a polyol method [37]. They observed an X-ray attenuation efficiency of 4.8 HU/mM at 65 kVp, higher than that of Iohexol (1.6 HU/mM).

#### 3.2.5. Yb (Z = 70)-Based NPs

Yb has a higher Z value and thus a higher X-ray attenuation coefficient than iodine (Z = 53) [4], making Yb-based NPs promising CT contrast agents.

Ghazanfari et al. investigated the X-ray attenuation properties of PAA-coated ultrasmall Yb_2_O_3_ NPs with an average particle diameter of 1.7 nm synthesized using a one-pot polyol method [32]. The PAA-coated ultrasmall Yb_2_O_3_ NPs showed good colloidal stability in aqueous media and nontoxicity in cellular cytotoxicity tests due to the PAA coating. The X-ray attenuation efficiency of the Yb_2_O_3_ NP suspension (6.8 HU/mM) was higher than that of Ultravist (4.4 HU/mM).

**Figure 9:**
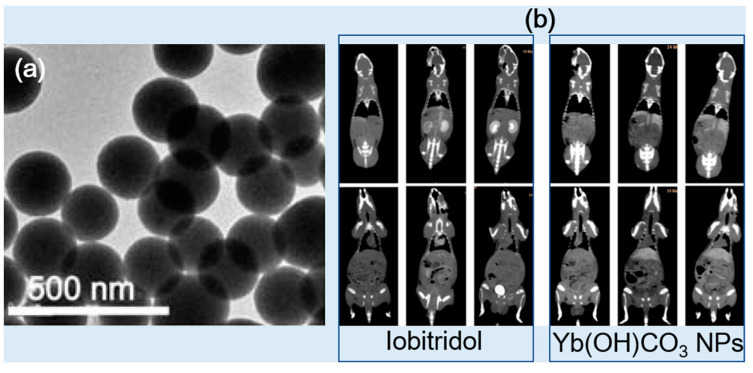
(**a**) TEM images of Yb(OH)CO_3_ NPs. (**b**) In vivo CT images of rats before and after the injection of Yb(OH)CO_3_ NPs (right) and Iobitridol (left) at 120 kVp [44].

Liu et al. synthesized Yb(OH)CO_3_ NPs (d = 170 nm) using a one-pot urea-based homogeneous precipitation method without using surface-coating ligands (Figure 9b) [44]. To measure CT contrast enhancement, the Yb(OH)CO_3_ NPs and Iobitridol were dispersed in PBS buffer containing 1% agarose with different Yb and I concentrations in a concentration range of 0–25 mg/mL. The Yb(OH)CO_3_ NPs exhibited an X-ray attenuation efficiency of ~9.0 HU/mM at 120 kVp, which was higher than that of Iobitridol (~3.6 HU/mM). In in vivo CT studies, the Yb(OH)CO_3_ NPs (injection dose: 1.0 mL of 50 mg Yb/mL) provided higher CT contrast in the liver (bottom, right) than Iobitridol (bottom, left) (injection dose: 0.3 mL of 350 mg I/mL) after intravenous injection at 120 kVp (Figure 9b), demonstrating the potential of the Yb(OH)CO_3_ NPs as CT contrast agents. On the contrary, Iobitridol exhibited contrasts at the kidneys (top, left) and bladder (bottom, left), whereas the Yb(OH)CO_3_ NPs did not (top and bottom, right). This is due to the primary accumulation of the Yb(OH)CO_3_ NPs in the liver due to their large particle size, whereas Iobitridol was quickly excreted through the kidneys and bladder as urine due to its molecular size.

Liu et al. synthesized oleic acid-coated NaYbF_4_:Er NPs (2.0% Er) (OA-UCNPs) (d = 40 nm) and modified them with phospholipid-polyethylene glycol terminated with carboxylic acid (PL-PEG-COOH) (or simply DSPE-PEG 2000) to obtain PEG-UCNPs for dispersibility in water [45]. The PEG-UCNPs exhibited an X-ray attenuation efficiency of ~9.9 HU/mM at 120 kVp, which was higher than that of Iobitridol (~3.4 HU/mM). In addition, the PEG-UCNPs exhibited high contrast in the heart, liver, spleen, and kidneys after intravenous injection at 120 kVp, whereas Iobitridol mostly accumulated in the urinary organs (kidney and bladder), and no contrast was detected in the heart, vessels, and other organs, indicating the short circulation time and rapid vascular permeation of Iobitridol.

Liu et al. synthesized PEGylated Er^3+^-doped (5 mol%) Yb_2_O_3_ upconversion NPs (PEG-UCNPs) (d = 170 nm) for use as high-performance contrast agents using a one-pot urea-based homogeneous precipitation process [46]. The potential of the PEG-UCNPs in upconversion fluorescence imaging and CT was investigated in vitro and in vivo. The NPs exhibited a long blood circulation time due to the PEG coating [20,21,22] and an X-ray attenuation efficiency of 10.0 HU/mM at 120 kVp, which was higher than that of Iobitridol (3.0 HU/mM). In vivo CT studies showed that after intravenous injection, the contrast enhancements for the liver were higher for the NP samples compared with that of Iobitridol. The PEG-UCNPs also showed contrast enhancements for the heart and spleen but not the kidneys due to their large particle size, whereas Iobitridol exhibited high contrast enhancement only for the kidneys due to its quick renal excretion due to its molecular size.

### 3.3. Other Heavy Metal-Based NPs (Ta, W, and Bi)

Other heavy metal-based NPs, such as Ta, W, and Bi, have been investigated as CT contrast agents because they possess atomic numbers higher than I, providing higher X-ray attenuation compared with commercial iodine contrast agents. In particular, Bi possesses the highest Z value among nonradioactive elements, thereby exhibiting very strong X-ray attenuation.

#### 3.3.1. Ta (Z = 73)-Based NPs

Ta is a highly biocompatible transition metal with negligible side effects in all redox states compared with other transition metals [95]. Thus, Ta has been extensively used in clinical implants, prosthetic joints, stents, and vascular clips for nearly 50 years. Ta has recently been used as a CT contrast agent because of its high X-ray attenuation coefficient [4].

Tantalum oxide (TaOx) NPs of uniform size were synthesized by Oh et al. using a simple microemulsion approach and surface-modified with V [47]. The particle diameter was controlled (6, 9, 13, and 15 nm) by varying the volume of ethanol in the synthesis. The X-ray attenuation efficiency of the TaOx NPs (~5.1 HU/mM at 100 keV) was obtained. The TaOx NPs (840 mg/kg) were injected intravenously into the tails of mice to observe the contrast enhancements for the liver, heart, kidneys, and spleen after injection. The contrast enhancements lasted for more than 3 h, indicating the prolonged circulation of the NPs in the bloodstream (attributable to the PEG coating) [20,21,22].

Bonitatibus et al. synthesized (2-diethylphosphato-ethyl)triethoxysilane-coated Ta_2_O_5_ NPs (d = ~6 nm and HD = 6 nm) with high stability in water [48]. The Ta_2_O_5_ NPs were synthesized via the controlled hydrolysis of tantalum ethoxide and then coated with (2-diethylphosphato-ethyl)triethoxysilane. At equal molar concentrations of tantalum and iodine, the NPs provided greater image contrast than Iopromide across the diagnostic X-ray spectrum between 80 and 140 kVp in in vitro phantom images. In addition, in vivo studies showed high contrast at the arterial system after intravenous injection into mice tails (1 mL of 0.92 M [Ta]) at 120 kVp. The same group improved their performance by synthesizing a zwitterionic siloxane polymer coating, which reduced the viscosity of the concentrated NP solutions by a factor of five, decreased the tissue retention of injected NPs by a factor of ten, and prevented pathological responses in the kidneys [49].

**Figure 10:**
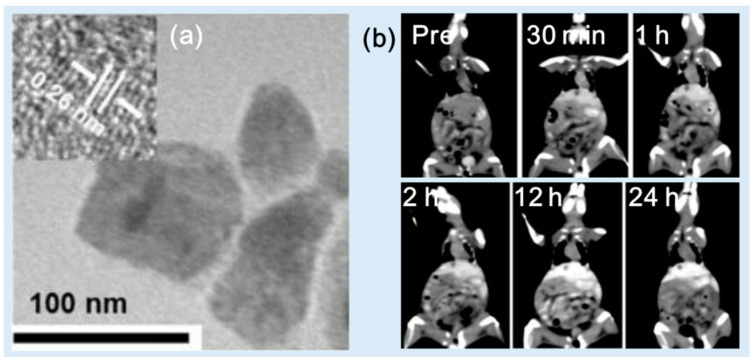
(**a**) TEM image of PEG-TaS_2_ NSs (the inset shows a magnified image showing the lattice fringe). (**b**) Time-dependent whole-body CT imaging of a mouse after the intravenous injection of PEG-TaS_2_ NSs at 120 kVp showing contrast enhancements at the liver [50].

Liu et al. synthesized 1,2-distearoyl-sn-glycero-3-phosphoethanolamine-N-methoxy(polyethylene glycol)-3000 (DSPE-PEG)-coated TaS_2_ NSs (PEG-TaS_2_ NSs) (d = 50–100 nm and HD = ~110 nm) for safe and efficient cancer treatment (Figure 10a) [50]. The TaS_2_ NSs were synthesized using the combinatorial grinding of bulk TaS_2_ and sonication processes and then coated with DSPE-PEG in aqueous media. The PEG-TaS_2_ NSs demonstrated high X-ray attenuation (6.3 HU/mM) at 120 kVp (similar to that of Iobitridol). After intravenous injection (4.5 mg Ta/kg), contrast enhancements at the liver (Figure 10b) were observed, demonstrating the potential of the NSs as CT contrast agents. The PEG-TaS_2_ NSs showed extended circulation times due to the PEG coating [20,21,22], making them useful for in vivo imaging and drug delivery, as well as cancer theranosis through the EPR effect.

#### 3.3.2. W (Z = 74)-Based NPs

Owing to the high Z value of W, W-based NPs have emerged as promising CT contrast agents due to their high X-ray attenuation properties [4]. Various W-based NPs and NRs have been investigated as X-ray contrast agents.

**Figure 11:**
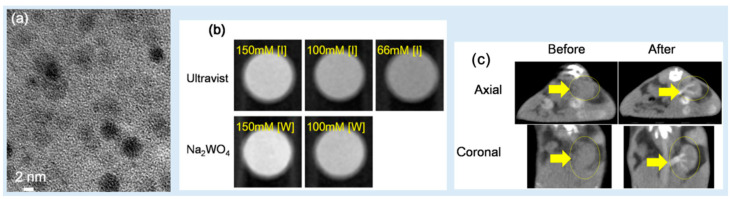
(**a**) HRTEM image of D-glucuronic acid-coated Na_2_WO_4_ NPs. (**b**) X-ray attenuation phantom images of the D-glucuronic acid-coated Na_2_WO_4_ NP solution at 70 kVp and Ultravist as a function of [I] and [W]. (**c**) Axial and coronal in vivo CT images of a mouse before and after the intravenous injection of D-glucuronic acid-coated Na_2_WO_4_ NPs into its tail. The arrows and circles serve to identify the kidney [51].

Kim et al. studied the use of _D_-glucuronic acid-coated Na_2_WO_4_ NPs as CT contrast agents [51]. The _D_-glucuronic acid-coated Na_2_WO_4_ NPs were synthesized through using a one-pot polyol method. The average particle diameter of the NPs was 3.2 nm (Figure 11a). In vitro phantom images showed stronger X-ray attenuation than that obtained using Ultravist at 70 kVp (Figure 11b); the X-ray attenuation efficiency of the NPs was ~10 HU/mM, whereas that of Ultravist was ~4.5 HU/mM. In addition, in vivo CT images showed contrast enhancements at the kidneys after intravenous injection (0.1 mmol W/kg) at 70 kVp (Figure 11c). This dose is much lower than that of the iodine contrast agents (2–6.4 mmol I/kg), indicating that the _D_-glucuronic acid-coated Na_2_WO_4_ NPs can be used as CT contrast agents.

Tian et al. synthesized polyvinyl pyrrolidone (PVP)-coated rubidium tungsten bronze (Rb_x_WO_3_) NRs (d × ℓ = 5 × 20–40 nm) for CT imaging [52]. The Rb_x_WO_3_ NRs were synthesized using a hydrothermal method. The WO_3_ precursor Rb_2_SO_4_ was mixed with polyvinyl pyrrolidone in ethylene glycol at 180 °C for 16 h. The NRs showed excellent contrast efficacy for CT imaging, as evidenced by an X-ray attenuation efficiency of ~7.1 HU/mM at 70 kVp, which was much higher than that of Ultravist (1.7 HU/mM). The CT signal at the tumor site was apparent immediately after intratumoral injection (3 mg/mL, 20 μL) at 70 kVp, indicating that the Rb_x_WO_3_ NRs can be used as contrast agents for in vivo CT imaging.

Zhou et al. produced tungsten oxide (WO_2.9_) NRs (length × diameter = 13.1 × 4.4 nm) via a facile thermal decomposition method [53]. The NRs modified with PEG exhibited water solubility and biocompatibility, as well as a higher X-ray attenuation efficiency (1.9 HU/mM) than Iohexol (0.5 HU/mM) at 80 kVp. They used the PEGylated WO_2.9_ NRs for in vivo tumor imaging and photothermal therapy by intratumorally injecting the sample solution into nude mice with HeLa tumors (20 mg W/kg). After injection, high-contrast enhancements at the tumor site were observed at 60 kVp. In addition, tumor ablation was achieved by employing photothermal therapy at 980 nm irradiation, implying that the NRs can be used as a theranostic agent in the treatment of tumors (i.e., CT diagnosis and photothermal tumor therapy).

Jakhmola et al. synthesized WO_3_ NPs as a CT contrast agent by stirring WCl_6_ in benzyl alcohol at 100 °C for 2 days. The biodegradable polymer poly-caprolactone (PCL) and PEG were used to coat the WO_3_ NPs (thickness w = 5–10 nm × d = 30–100 nm and HD = ~100–200 nm) [54]. Because the PCL layer prevents particle aggregation, the PCL/PEG-coated WO_3_ NPs showed good stability and nontoxicity. The PCL/PEG-coated WO_3_ NPs had an X-ray attenuation efficiency of ~15 HU/mM at 49 kVp, which was nearly four times greater than that of the commercial iodine contrast agent Fenestra VC. After intravenous injection (0.73 mmol W/kg mice), contrast enhancements were observed at the heart, liver, spleen, and kidneys at 50 kVp and then decayed with time, finally reaching the initial contrasts ~10 h after the injection. The PCL/PEG-coated WO_3_ NPs had extended blood circulation times due to the PEG coating [20,21,22], making them ideal for angiography, but were rapidly excreted from the body, minimizing long-term toxicity.

Dong et al. synthesized biocompatible and high-performance amino acid-capped MnWO_4_ NRs (d = 20 nm × ℓ = 50 nm) using a hydrothermal method [55]. The NRs exhibited an X-ray attenuation efficiency of 4.5 HU/mM at 120 kVp, which was higher than that of Iobitridol (3.0 HU/mM). In in vivo experiments, contrast enhancements at the liver and kidneys were observed after intravenous injection (1 mL of 0.42 M [W]) at 120 kVp. The amino acid-capped MnWO_4_ NRs showed sustained contrast enhancement at the liver owing to their accumulation, making them useful for the detection of liver diseases. In addition, the NRs functioned as a T_1_ MRI contrast agent owing to Mn, demonstrating their potential as a T_1_ MRI-CT dual imaging agent.

#### 3.3.3. Bi (Z = 83)-Based NPs

Among nonradioactive elements, Bi possesses the highest atomic number and thus the highest X-ray attenuation coefficient [4], making Bi-based NPs extremely useful as a CT contrast agent. From polonium (Po) (Z = 84) onward, all elements are radioactive and therefore not suitable for biomedical applications. In addition, Bi is a relatively inexpensive heavy metal with low toxicity.

**Figure 12:**
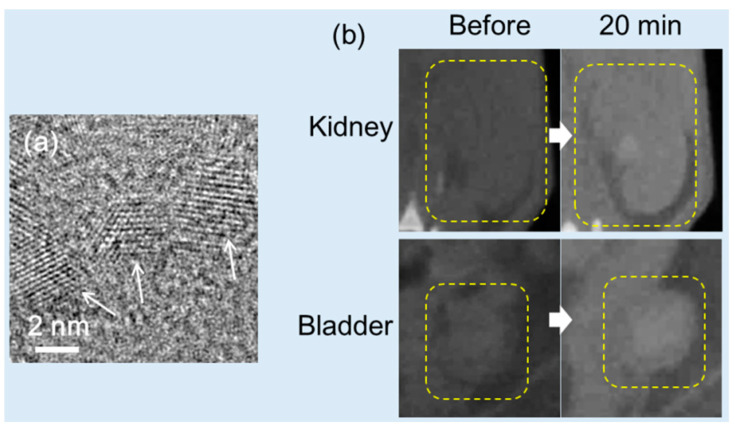
(**a**) HRTEM image of PAA-coated ultrasmall Bi_2_O_3_ NPs (the arrows indicate NPs). (**b**) In vivo CT images of a mouse kidney and bladder before and 20 min after the intravenous injection of the suspension of PAA-coated ultrasmall Bi_2_O_3_ NPs into the mouse’s tail (indicated by the dotted squares) [32].

Ghazanfari et al. synthesized PAA-coated ultrasmall Bi_2_O_3_ NPs (d = 2.3 nm) (Figure 12a) using a one-pot polyol method and examined their X-ray attenuation properties [32]. The NPs exhibited good colloidal stability and nontoxicity in in vitro cellular assays due to the PAA coating. The X-ray attenuation efficiency of the Bi_2_O_3_ NP suspension (11.7 HU/mM) at 70 kVp was greater than that of Ultravist (4.4 HU/mM). To demonstrate their effectiveness as a CT contrast agent, the suspension of the PAA-coated ultrasmall Bi_2_O_3_ NPs was injected into the mouse tail vein (dose = ~0.1 mmol/kg), which is significantly smaller than the average injection dose used for iodine contrast agents (1.5 mmol/kg). CT images were taken before and after the injection, and positive contrasts were observed for the mouse kidney and bladder after injection, as illustrated in Figure 12b. The contrast enhancement at the liver was minimal because of the low injection dose. However, contrast enhancements at the kidney and bladder were achieved even at the low injection dose due to the excretion of the NPs through the renal system. The results suggested the need for ultrasmall CT contrast agents for practical applications. The same group synthesized D-glucuronic acid-coated BiOI NPs using a one-pot polyol method [82]. The particle diameter was controlled by varying the solvent volume so that 1.9- and 6.1 nm BiOI NPs were produced using 20 and 10 mL of triethylene glycol, respectively. A D-glucuronic acid-coated BiOI NP (d = 1.9 nm) solution was used to determine their X-ray attenuation efficiency using in vitro phantom imaging. The sample solution exhibited exceptionally high X-ray attenuation owing to the combined effects of Bi and I. It exhibited ~21 HU/mM, which was 4.4 times higher than that of Ultravist (~4.8 HU/mM) and ~127 times higher at the same number density, highlighting the potential of D-glucuronic acid-coated BiOI NPs as CT contrast agents.

Rabin et al. synthesized bismuth sulfide (Bi_2_S_3_) NSs (d × w = 10–50 nm × 3–4 nm) via precipitation in the presence of 3-mercaptopropionic acid [84]. The NSs were coated with polyvinylpyrrolidone (PVP) for stability in aqueous media and biocompatibility. This formulation showed a longer in vivo circulation time (140 min), higher stability, and a five-fold increase in the X-ray attenuation coefficient (~9.7 HU/mM), compared to that of Iopromide at 50 kVp. The lymph nodes of mice were visible after intravenous injection (dose = 11.4 μmol Bi) into Balb/c mice, demonstrating that the Bi_2_S_3_ NSs could be an excellent CT contrast agent for disease diagnosis. In addition, the PVP-coated Bi_2_S_3_ NSs exhibited a long blood circulation time, making them suitable for obtaining CT scans for extended periods.

Swy et al. synthesized 38 nm bismuth(0) NPs encapsulated within 120 nm poly(DL-lactic-co-glycolic acid) (PLGA) NPs using an oil-in-water emulsion methodology [86]. The PLGA-coated Bi NPs exhibited an X-ray attenuation coefficient of 10.2 HU/mM at 80 kVp in aqueous media.

Wei et al. developed oligosaccharide-modified Bi-NPs (OS-Bi-NPs) (d = 22 nm) as a CT contrast agent for GIT imaging [87]. The Bi-NPs were synthesized in 1-dodecanethiol under argon flow at 178 °C to avoid air oxidation and coated with sweet OS to make OS-Bi-NPs that were nontoxic and stable in aqueous media and facilitate their oral administration. The X-ray attenuation efficiency of the OS-Bi-NPs (i.e., 8.5 HU/mM at 80 kVp and 6.4 HU/mM at 120 kVp) was 1.5 times greater than that of the conventional clinical CT contrast agent BaSO_4_ used for GIT imaging. In addition, the OS-Bi-NPs were employed in GIT imaging in vivo (dose = 400 μL of 14 mg Bi/mL) at 80 kVp. Small intestinal loops were observed, implying the feasibility of the diagnosis of inflammatory, neoplastic intestinal lesions and incidental extra-intestinal pathological changes.

Because of their chemical stability, dual chalcogenides are frequently used as CT contrast agents. Mao et al. obtained ultrasmall Bi_2_Se_3_ nanodots (2.7 nm) stabilized with BSA (BSA-Bi_2_Se_3_ nanodots), which were synthesized via the reaction of hydroxyethylthioselenide with bismuth chloride in an aqueous solution under ambient conditions [88]. BSA was used to stabilize the Bi_2_Se_3_ nanodots in aqueous media, which were further conjugated with radioactive ^99m^Tc for the single-photon-emission CT of cancer. They found that the X-ray absorption coefficient was approximately 7.06 HU/mM at 55 kVp, higher than that of Iopromide (3.9 HU/mM). The BSA-Bi_2_Se_3_ nanodots exhibited contrast enhancements at the 4T1 tumor of mice after intratumoral injection (dose = 50 μL, 0.078 mg Bi_2_Se_3_) at 55 kVp. The CT value at the tumor was significantly enhanced from 51 HU to 467 HU after injection. In addition, the high potential of the BSA-stabilized Bi_2_Se_3_ nanodots as theranostic agents was demonstrated due to their excellent performance in PA imaging, photothermal therapy, and radiation therapy for tumors.

## 4. Cytotoxicity

For applications as CT contrast agents, surface-modified heavy metal-based NPs in pure metallic or chemical forms (metal = Pd, Ag, Ce, Gd, Dy, Ho, Yb, Ta, W, Pt, Au, and Bi) must be nontoxic. In addition, metal or metal ion leaking must be avoided as heavy metals and heavy metal ions are generally toxic depending on the type of metal and metal ion species [95,96,97,98,99,100,101,102,103,104,105]. Au and Ta are used in tooth crown and dental implants, respectively, and are less toxic than other metals. Gd(III) ions are toxic and can cause nephrogenic systemic fibrosis (NSF), a fibrosis of the skin and internal organs [100]. Therefore, heavy metal-based NPs must be completely coated with hydrophilic and biocompatible ligands to avoid metal and metal ion leaking.

**Figure 13:**
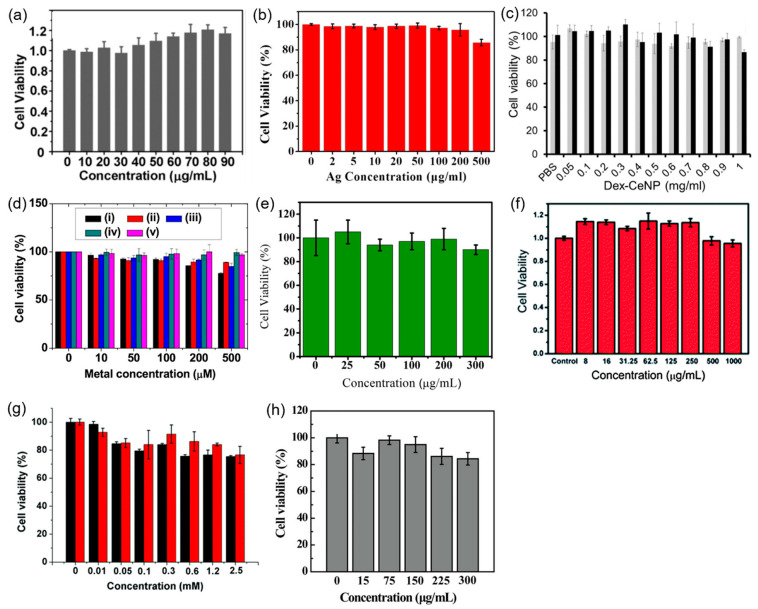
In vitro cell viabilities of (**a**) PEG-coated porous Pd NPs in human lung carcinoma cells (A549) [106]; (**b**) albumin-stabilized Ag nanodots in human oral epithelial cells (KB) [28]; (**c**) dextran-coated CeO_2_ NPs in human liver cancer cells (HepG2) (incubation time: gray = 1 h and black = 24 h) [29]; (**d**) PAA-coated ultrasmall (i) Gd_2_O_3_, (ii) Yb_2_O_3_, (iii) Dy_2_O_3_, (iv) Bi_2_O_3_, and (v) NaTaO_3_ NPs in human prostate cancer cells (DU145) [32]; (**e**) PEG-coated HoF_3_ NPs in mouse fibroblast cells (L929) [38]; (**f**) amino acid-coated MnWO_4_ NRs in human embryonic kidney (HEK) 293 cells [55]; (**g**) Pt@BSA nanocrystals in A549 cells (incubation time: black = 24 h and red = 48 h) [56]; (**h**) PEG-PEI-coated Au NPs in KB cells [68].

Surface-modified heavy metal-based NPs must be nontoxic for in vitro and in vivo applications. To demonstrate this, the in vitro cell viabilities of heavy metal-based NPs that exhibit very low cellular toxicities are provided in Figure 13a–h [28,29,32,38,55,56,68,106]. The PEG-coated porous Pd NPs that correspond to Figure 13a were incubated in human lung carcinoma cells (A549) for 24 h and exhibited nontoxicity up to 90 μg/mL [106]. The albumin-stabilized Ag nanodots that correspond to Figure 13b exhibited little cytotoxicity in human oral epithelial cells (KB) after incubation for 24 h; a cell viability above 85% was maintained for nanodot concentrations up to 500 μg Ag/mL [28]. The dextran-coated CeO_2_ NPs that correspond to Figure 13c showed little cytotoxicity in human liver cancer cells (HepG2) after incubation for 1 and 24 h; no substantial toxicity was observed for concentrations up to 1 mg NP/mL [29]. After incubation for 24 h, PAA-coated ultrasmall Gd_2_O_3_, Yb_2_O_3_, Dy_2_O_3_, Bi_2_O_3_, and NaTaO_3_ NPs exhibited very low cytotoxicity in human prostate cancer cells (DU145) for up to 500 μM [X] (X = Gd, Yb, Dy, Bi, and Ta) (Figure 13d) [32]. PEG-coated HoF_3_ NPs exhibited very low cytotoxicity in mouse fibroblast cells (L929) after incubation for 24 h; the cell viability was above 85% for up to 300 μg/mL of the NPs (Figure 13e) [38]. Amino acid-coated MnWO_4_ NRs exhibited almost no toxicity in 293 human embryonic kidney (HEK) cells after incubation for 24 h even up to a high concentration of 1000 μg/mL; the cell viability remained above 90% (Figure 13f) [55]. BSA-loaded Pt (Pt@BSA) nanocrystals exhibited low cytotoxicity in A549 cells up to 2.5 mM Pt after incubation for 24 and 48 h; the cell viability was above 80% up to 2.5 mM Pt, and the cell viability slightly increased after 48 h of incubation due to BSA, which promoted cell growth (Figure 13g) [56]. PEG-PEI-coated Au NPs exhibited very low cytotoxicity in KB cells for up to 300 μg/mL after 24 h of incubation (Figure 13h) [68]. Therefore, all surface-modified heavy metal-based NPs are nontoxic and suitable for in vitro and in vivo CT imaging applications.

## 5. Conclusions and Perspectives

Heavy metal-based NPs exhibit comparable or higher X-ray attenuation efficiencies compared with their commercial iodine-based counterparts [5,6,7,8,9,10,11]. Side effects such as acute kidney disease resulting from the high injection doses (1–2 mmol/kg) of commercial iodine-based CT contrast agents [13] may be mitigated using heavy metal-based NPs that possess X-ray attenuation efficiencies higher than those of iodine contrast agents. In this review, the applications of Pd-, Ag-, Ce-, Gd-, Dy-, Ho-, Yb-, Ta-, W-, Pt-, Au-, and Bi-based NPs as CT contrast agents were discussed, as demonstrated via in vitro phantom and in vivo CT images.

Heavy metals can strongly attenuate X-rays as X-ray attenuation is proportional to atomic number (Z) [4], making them more desirable than molecular iodine contrast agents, which possess only three iodines per molecule. In addition, they exhibit longer blood circulation times and, consequently, longer imaging times compared with iodine contrast agents. Furthermore, they can be easily surface-functionalized with cancer-targeting ligands, drugs, and other imaging agents, enabling advanced applications [15,16]. Heavy metal-based NPs also possess extremely useful magnetic, optical, and therapeutic properties. Therefore, they can be used as multimodal imaging or theranostic agents.

The advantages and disadvantages of heavy metal-based NPs, potential side effects, and physicochemical properties affecting NP accumulation or renal excretion are summarized in Table 2. Based on this summary, we suggest that further research is necessary to develop heavy metal-based NPs for use as CT contrast agents.

Despite promising developments in the deployment of heavy metal-based NPs as CT contrast agents, a few challenges remain unaddressed. NP toxicity is one of the major problems. Although numerous studies have shown very low NP toxicity via in vitro cellular assays and in vivo histology assays, more research must be conducted to fully understand their long-term behavior and potential side effects due to their accumulation in tissues and organs. One effective approach to solve the toxicity problem is to make NPs completely excretable via the renal system within a few hours. To this end, the core and hydrodynamic diameters of NPs must be smaller than ~3 nm [18,19]. In addition, their colloidal stability must be improved because aggregated NPs weakly contribute to X-ray attenuation compared with well-dispersed NPs. In addition, aggregated NPs accumulate in tissues and organs, causing toxicity. Therefore, synthetic strategies must be developed to address the long-term toxicity and renal excretion of NPs for in vivo applications.

## Figures and Tables

**Table 1:** X-ray attenuation efficiencies (η) of various heavy-metal based NPs, nanorods (NRs), and nanosheets (NSs).

Metal	Z ^1^	Chemical Formula	Coating Ligand	Key Finding/Application	Morphology, Size (nm) ^2^	η (HU/mM)	Tube Voltage (kVp)	Ref
Pd	46	FePd	Cysteamine	CT/MRI/photoacoustic tri-modal imaging probe	Spherical, 3.4	2.6	-	[24]
Ag	47	Ag	Generation-5 (G5) poly(amido-amine) dendrimer	Particle diameter-dependent X-ray attenuation efficiency	Spherical, 8.8 12.4 16.1 23.2	~2.4 ~4.6 ~7.2 ~4.0	80	[25]
		Ag	Hyaluronic acid (HA)	SPECT ^3^ lung cancer imaging in vivo using ^99m^Tc-HA-Ag NPs	Spherical, ~10	3.5	-	[26]
		Ag_2_S	Glutathione	85% renal excretion within 24 h and nearly particle size-independent X-ray attenuation efficiency	Spherical, 2.3, 3.1, 5.1	~3.5 ~2.7 ~2.3 ~1.9	80 100 120 140	[27]
		Ag	Bovine serum albumin	Useful for CT probe and photothermal cancer therapy agent	Spherical, 5.8	5.7	-	[28]
Ce	58	CeO_2_	Dextran	Inflammatory bowel disease imaging under oxidative damage protection	Spherical, 4.8	~6.3 ~4.8 ~3.8 ~3.2	80 100 120 140	[29]
		CeO_2_	Murine serum albumin	Long-time imaging of organs and tumor	Spherical, 5.1	-	-	[30]
Gd	64	Gd_2_O_3_	5-amino-2,4,6-triiodoisophthalic acid	MRI/CT dual imaging in vivo application	Spherical, 2	11.8	70	[31]
		Gd_2_O_3_	Polyacrylic acid	Ultrasmall NPs and high X-ray attenuation efficiency	Spherical, 1.9	5.9	70	[32]
		Gd(IO_3_)_3_H_2_ O	_D_-glucuronic acid	Properties useful as MRI/CT dual imaging agent	Mixture of nanosheet & nanorod, 110, 750 (nanosheet); 325 × 150 (nanorod)	~5.1	35	[33]
		GdF_3_:Fe	Polyethylene glycol	In vivo MRI/CT dual imaging application	Nanorod, 51.9 × 31.3	6.9	120	[34]
		GdF_3_	Polyacrylic acid	Properties useful as MRI/CT dual imaging agent	Nanoplate, 10.6 × 7.0 × 4.2	~7.9	60	[35]
Dy	66	Dy_2_O_3_	Polyacrylic acid	Ultrasmall NPs and high X-ray attenuation efficiency	Spherical, 1.8	6.1	70	[32]
		Dy_2_O_3_	Polyethyleneimine	High X-ray attenuation efficiency suitable as CT contrast agent	Spherical, 79–102	~5	120	[36]
		DyVO_4_	Polyacrylic acid	Properties useful as MRI/CT dual imaging agent	Spherical, 60	4.8	65	[37]
Ho	67	HoF_3_	Polyethylene glycol	In vivo MRI/CT dual imaging application in tumor diagnosis	Spherical, 38	190	120	[38]
		BaHoF_5_	Polyethylene glycol	CT/CT angiography/CT perfusion and ischemic stroke imaging	Spherical, 7	4.8	80	[39]
		NaHoF_4_	Polyethylene glycol	In vivo MRI/CT dual imaging and tumor imaging	Spherical, 3.2	6.9	120	[40]
		HoVO_4_	Polyacrylic acid	Properties useful as MRI/CT dual imaging agent	Spherical, 65	4.8	65	[37]
Yb	70	Yb_2_O_3_	Polyacrylic acid	Ultrasmall NPs and high X-ray attenuation efficiency	Spherical, 1.7	6.8	70	[32]
		Yb_2_O_3_	_D_-glucuronic acid	Ultrasmall NPs and high X-ray attenuation efficiency	Spherical, 2.1	~9.7	70	[41]
		BaYbF_5_	-	CT contrast agent for osteochondral interface imaging	Spherical, 8, 11	~2.7 (8 nm), ~2.6 (11 nm)	70	[42]
		BaYbF_5_@SiO_2_	-	CT contrast agent for osteochondral interface imaging	Spherical, 27, 34	~1.8 (27 nm), ~1.2 (34 nm)	70	[42]
		Yb	3-mercaptopropionic acid	Applicable as CT/spectral photon-counting CT contrast agent	Spherical, 4.75	~10.4	55	[43]
		Yb(OH)CO_3_	-	A large scale synthesis and in vivo CT application	Spherical, 170	~9.0	120	[44]
		NaYbF_4_:Er	Phospholipid-polyethylene glycol	Long circulation time and high contrasts in in vivo CT images	Spherical, 40	~9.9	120	[45]
		Yb_2_O_3_:Er	Polyethylene glycol	Long circulation time and in vivo CT/upconversion optical dual imaging	Spherical, 170	10.0	120	[46]
Ta	73	NaTaO_3_	Polyacrylic acid	Ultrasmall NPs and high X-ray attenuation efficiency	Spherical, 1.5	10.3	70	[32]
		TaO_x_	Polyethylene glycol-silane	Large-scale synthesis and in vivo CT/optical dual imaging through rhodamine-B-isothiocyanate conjugation	Spherical, 6, 9, 13, 15	~5.1 (6 nm)	100	[47]
		Ta_2_O_5_	(2-diethylphosphatoethyl)triethoxysilane	In vivo CT application to arterial system in high resolution	Spherical, ~6	-	-	[48]
		TaS_2_	1,2-distearoyl-sn-glycero-3-phosphoethanolamine-N-[methoxy(polyethylene glycol)-3000	In vivo CT-guided chemo-photothermal cancer therapy	Nanosheet, 50–100	6.3	120	[50]
W	74	Na_2_WO_4_	_D_-glucuronic acid	Ultrasmall NPs and in vivo CT application	Spherical, 3.2	~10	70	[51]
		Rb*_x_*WO_3_	Polyvinyl pyrrolidone	In vivo CT-guided chemo-photothermal cancer therapy	Nanorod, 5 × 20–40	~7.1	70	[52]
		WO_2.9_	Polyethylene glycol	In vivo tumor CT imaging and photothermal therapy	Nanorod, 4.4 × 13.1	1.9	80	[53]
		WO_3_	Poly-caprolactone and polyethylene glycol	Long circulation time up to 3 h and in vivo CT application	2D platelet, 30–100 × 5–10	~10	49	[54]
		MnWO_4_	Amino acid	In vivo CT/MRI dual imaging application	Nanorod, 20 × 50	~4.5	120	[55]
Pt	78	Pt	Bovine serum albumin	Long circulation time and in vivo CT application	Spherical, 2.1	16.8	120	[56]
		Pt	Polyacrylic acid, poly(acrylic acid-co-maleic acid), poly(methyl vinyl ether-alt-maleic acid)	Ultrasmall NPs and high X-ray attenuation efficiency	Spherical, 2.0	16.4 18.4	50 70	[57]
		Pt	Mercaptoaminopolyglycol-chlorin e6	In vivo CT/photoacoustic imaging-guided photothermal cancer therapy	Mesophorous, 70	3.1	120	[58]
		Pt	Polyethylene glycol	In vivo CT/chemotherapy/photothermal cancer therapy	Mesophorous, 94	5.5	120	[59]
		Pt	Poly(maleic anhydride-alt-1-octadecene)– polyethylene glycol	Long blood circulation time/in vivo CT/photoacoustic imaging/photothermal therapy/radoatherapy of cancer	Nanoworm, ~3 × ~10	3.9	-	[60]
		Pt	Extract from Prosopis farcta fruits	Green Pt NP synthesis	Spherical, 3.8	6.6	80	[61]
		Pt	Human serum albumin	In vivo CT/photoacoustic imaging/photothermal cancer therapy	Spherical, 6.7	~5.6	-	[62]
		Pt	Polyethylene glycol	Higher photothermal conversion efficiency than Pt NPs and in vivo CT-guided photothermal cancer therapy	Hollow cube, 30	~5.39	-	[63]
Au	79	Au	Polyethylene glycol	Long blood circulation time (>4 h) and in vivo hepatoma CT imaging application	Spherical, 31	5.0	120	[64]
		Au	Polyethylene glycol	Application to in vivo blood pool imaging	Spherical, 10	4.8	50	[65]
		Au	Mercaptosuccinic acid	No particle size dependent X-ray attenuation efficiency	Spherical, 4.7, 13.2, 35.0, 76.4	10.6 13.0	70 45	[66]
		Au	Bovine serum albumin	In vitro CT imaging and chemotherapy of lung cancer cells	Spherical, 11.2	~5.6	120	[67]
		Au	Polyethylene glycol-polyethyleneimine	Application to in vivo blood pool CT imaging and tumor imaging	Spherical, 1.9, 2.9, 3.9, 4.6	~9 (2.9 nm)	-	[68]
		Au	Lactobionic acid	In vivo CT imaging of cancer	Spherical, 2.7	8.5	80	[69]
		Au	G5-poly(amidoamine) dendrimer	Application in vivo CT imaging	Spherical, 1.9, 2.8, 4.0	9.8 (4.0 nm)	80	[70]
		Au	NH_2_- fluorescein isothiocyanate-(polyethylene glycol- α-tocopheryl succinate)-(polyethylene glycol- folic acid) G5-dendrimer	In vivo targeted CT imaging and cancer therapy	Spherical, 3.3	~6.0	80	[71]
		Au	Gum Arabic	Large amounts accumulating in the liver, lung, and spleen in in vivo biodistribution	Spherical, 15–20	~4.9	80	[72]
		Au	Folic acid-conjugated silica	In vivo tumor tumor targeting CT imaging and in vitro radiation/photothermal therapy of cancer cells	Nanorod, 17.8 × 46.0	4.9	-	[73]
		Au	Glycol chitosan	Improved tumor accumulation and in vivo CT imaging of liver cancer	Spherical, 24	~2.8	70	[74]
		Au	Diatrizoic acid- Aptamer	In vivo tumor location via CT and fluorescence-guided resection of tumor	Spherical, 2.4	8.2	-	[75]
		Au	Gum Arabic	Green synthesis of colloidally stable Au NPs by laser ablation in aqueous solution	Spherical, 1.85	~4.3	80	[76]
		AuAg (3:1)	Folic acid-G5 poly(amidoamine) dendrimer	Targeted CT imaging of cancer cells in vitro	Spherical, 13.4	~6.3	100	[77]
		Au	Polyethylene glycol	Nearly particle size-independent X-ray attenuation efficiency and particle size-dependent biodistribution	Spherical, 3.9, 14.8, 50.6, 78.9, 99.2, 152.3	4.0–4.2	80	[78]
		Au	Cathepsin	Particle size-dependent in vivo accumulation/CT contrast at the tumor such that 10 nm > 30 nm > 100 nm	Spherical, 10, 30, 100	25.4 22.0	35 85	[79]
		Au	Heparin–amino acid 3,4-dihydroxyphenylalanine	Liver-specific CT imaging agent	Spherical, 24.0	21.9	70	[80]
Bi	83	Bi_2_O_3_	Polyacrylic acid	Ultrasmall NPs, high X-ray attenuation efficiency, and in vivo CT imaging	Spherical, 2.3	11.7	70	[32]
		BiOI	_D_-glucuronic acid	Ultrasmall NPs and very high X-ray attenuation efficiency	Spherical, 1.9	~21	70	[82]
		BiOI	Polyvinyl pyrrolidone	Very high X-ray attenuation efficiency	Spherical, 2.8	~20	75	[83]
		Bi_2_S_3_	Polyvinyl pyrrolidone	High X-ray attenuation efficiency, long circulation time of >2 h, and in vivo CT imging	Nanosheet, 10–50×3–4	~9.7	50	[84]
		Bi	1,2-propanediol and glucose	High payload element Bi NP CT contrast agent	Faceted, 74	~5.9	80	[85]
		Bi	Poly(DL-lactic-co-glycolic acid)	Potential agent for dual modality fluorescence and CT imaging	Spherical, 38	10.2	80	[86]
		Bi	Oligosaccharide	Simple synthesis of Bi NPs for in vivo gastrointestinal CT imaging	Spherical, 22	8.5 6.4	80 120	[87]
		Bi_2_Se_3_	Bovine serum albumin	In vivo CT/photoacoustic imaging-guided synergetic radiophotothermal therapy of cancer	Spherical, 2.7	7.06	55	[88]

^1^ Z: atomic number. ^2^ Size: nanoparticle diameter = d; nanorod = d × ℓ; nanosheet or nanoplate = d × w or d × ℓ × w (d: diameter; ℓ: length; w: thickness). ^3^ SPECT: single-photon emission computed tomography. G5: Generation 5 of dendrimers.

**Table 2:** The advantages and disadvantages of heavy metal-based NPs, as well as the potential side effects and physicochemical properties affecting NP accumulation or renal excretion.

Subject	Description
Advantages	(1) Higher X-ray attenuation, leading to the improved visualization of anatomical structures and abnormalities. (2) Longer blood circulation times, allowing for extended CT scans and providing improved image quality through EPR effects. (3) Targeted imaging through easy conjugation with specific ligands or antibodies, allowing for the sensitive detection of diseases. (4) Drug delivery, allowing for chemotherapy. (5) Additional optical, magnetic, and antibacterial properties, allowing for multimodal imaging and imaging-guided therapy.
Disadvantages	(1) Poor renal excretion of large NPs (d > 3 nm), leading to long-term toxicity owing to their accumulation in the body. (2) Heavy metal-based NPs are generally expensive. (3) Limited knowledge on NP contrast agents.
Potential side effects	(1) The side effects depend on metal species (Pd, Ag, Ce, Gd, Dy, Ho, Yb, Ta, W, Pt, Au, and Bi) [96,97,98,99,100,101,102,103,104,105]. (2) NPs should be completely grafted with hydrophilic and biocompatible ligands and excreted through the renal system to minimize potential side effects.
Physicochemical properties affecting NP accumulation or renal excretion	(1) Smaller NPs (d < 3 nm) can be excreted via the renal system. (2) Smaller surface-coating ligands are better for renal excretion. (3) Zwitterionic ligands provide better renal excretion. (4) Positively charged NPs may bind to negatively charged cell membranes or proteins, thereby delaying NP excretion. (5) The renal excretion of NRs is more difficult than the renal excretion of spherical NPs.

## Data Availability

Data sharing is not applicable.
